# Type 1 neurodegeneration with brain iron accumulation: a case report

**DOI:** 10.1186/s13256-022-03430-7

**Published:** 2022-06-03

**Authors:** Pedro Enrique Labrada-Aguilera, David Armando Guach-Hevia, Carlos Rafael Almira-Gómez, Daniel Alejandro González-Lago

**Affiliations:** 1 “Lucia Iñiguez Landin” Hospital, Holguin, Cuba; 2 Medical Sciences University, Holguin, Cuba; 3 “Pedro Diaz Coello” Policlinic, Holguin, Cuba

**Keywords:** Neurodegeneration, “Eye of the tiger” sign, Pantothenate kinase, Brain iron accumulation, Cuba, Case report

## Abstract

**Background:**

Type 1 neurodegeneration with brain iron accumulation is a rare neurological disorder with estimated prevalence of one to two per million persons worldwide, characterized by progressive degeneration of basal ganglia, globus pallidus, and reticular part of substantia nigra, produced by brain iron accumulation due to a defect in the gene producing pantothenate kinase 2. Clinical presentations include dystonia, dysarthria, dysphagia, dementia, severe mental retardation, and severe movement disability at later stages. The characteristic pattern on brain magnetic resonance imaging shows the “eye of the tiger” sign. Treatment in late stages is mainly symptomatic. We report the case of a Cuban boy with high-severity brain iron accumulation, with positive clinical and imaging findings diagnosed in a late stage of the illness. This degree of severity has never been reported in Cuba and is rarely reported worldwide.

**Case presentation:**

We present the case of a 19-year-old male white Cuban boy who presented to our department with features of spasticity, dystonia, gait difficulty, dysarthria, dysphagia, aggressiveness, and sleep disorders. He was diagnosed with pantothenate kinase-associated neurodegeneration on the basis of clinical findings and typical “eye of the tiger” pattern on brain magnetic resonance imaging. Detailed evaluation was carried out, and symptomatic treatment and physiotherapy were started with trihexyphenidyl, cabergoline, baclofen, and intramuscular botulinum neurotoxin as well as daily home sessions of passive stretching, weight bearing, and muscle massaging. At 3 months reevaluation, the patient showed a great improvement of motor function, with a decrease of dystonic symptoms, although language, cognition, and functional independence showed no improvement. The prognosis of the patient remains reserved.

**Conclusion:**

The diagnosis can be made based on the presence of clinical and imaging features. The presence of “eye-of-the-tiger” sign on magnetic resonance imaging must be considered a nearly pathognomonic sign of neurodegeneration with brain iron accumulation presence. Treatment after high-severity presentation remains directed toward symptomatic findings. Both dopamine agonists and anticholinergic agents are useful to treat motor symptoms, but there is not yet an effective treatment to stop the underlying degeneration. New therapeutic approaches are needed to counteract late stages of the disease and improve prognosis.

## Introduction

Type 1 neurodegeneration with brain iron accumulation (NBIA), also known as pantothenate kinase-associated neurodegeneration (PKAN), is an autosomal recessive disorder due to mutations in the gene producing PANK2 located in chromosome 20p13-p12.3, with estimated prevalence of one to two per million persons worldwide, and a highly variable phenotype [1]. PKAN is the most common disorder within the group of NBIA disorders, formerly known as Hallervorden–Spatz disease (HSD) [2]. The exact etiology of PKAN is not known. One proposed hypothesis is that abnormal peroxidation of lipofuscin to neuromelanin and deficient cysteine deoxygenase lead to abnormal iron accumulation in the brain, specifically at the globus pallidus and pars reticulata of substantia nigra, provoking both axonal and neuronal damage [3]. Motor manifestations of PKAN include dystonia, chorea, pyramidal signs, Parkinsonism, spasticity, dysarthria/anarthria, and dysphagia. Cognitive impairment, psychiatric features, progressive dementia, oculomotor deficits, and retinopathy may also be present [2]. The genotype–phenotype association is not well understood, and key features such as rate of progression, age at onset, and signs and symptoms are highly variable, even among siblings and individuals with identical mutations [4]. Treatment remains mostly symptomatic. A multidisciplinary team approach involving physical, occupational, and speech therapists may be needed to improve functional skills and quality of life. We report the case of a Cuban boy with high-severity brain iron accumulation, with positive clinical and imaging findings, diagnosed in a late stage of the disease. This degree of disease severity has never been reported in Cuba and is rarely reported worldwide. Symptomatic treatment and physiotherapy were prescribed. The prognosis of the patient remains reserved.

## Case description

A 19-year-old male white Cuban patient was brought to medical consultation by his mother, complaining of progressive rigidity of head and arms, resting tremor of hands, severe speech problems, and severe swallowing difficulties,that started around 4 months before. His mother also referred aggressiveness and sleep problems. Regarding personal history, the mother referred that the patient had appropriate neurologic development until 5 years old, then began to present progressive gait alterations with transitory extension postures of lower limbs, which first appeared with walking, then with motion intention, until becoming permanent. Several months later, he started presenting dystonic postures described as irreducible extension of head, lower limbs, and wrists, combined with inexpressive face. In the last year, functionality had been seriously affected, to the point of complete reliance for basic and instrumental daily activities. From that moment on, he has been confined to a wheelchair and bed. The patient had been never properly diagnosed because his family lived in a poor village in the mountains, and through the years his clinical state remained relatively constant, until about 4 months before, when he started to show worsening symptoms and her mother decided to take him to the provincial hospital, from where his case was referred to our service.

On examination, he showed incoherence in the interview, with dysarthria, low-tone voice, behavioral instability, and hetero-aggressiveness. He only tolerates liquid food, showing dysphagia to solids. Cognitive examinations showed average results, without progressive intellectual decline. Direct psychological interview/exploration showed impulsive behavior, impatience, decreased perception of reality, fear of social contacts, plane moral control, and moderate anxiety. When evaluating praxis through neuropsychological tests, the patient was capable of completing simple orders but could not achieve those with greater complexity, related to memory and motor control.

On neurological examination, the patient showed inexpressive but symmetric face, with limited upper ocular movements, with tongue contracted in dystonic posture. When the patient tried to move, he mobilized his four extremities, showing dystonic posture in extension of the limbs and head. Both muscular and tendinous reflexes appeared diminished. Sole reflexes were normal. Neuroconductive and electromyographic investigations showed no alterations. A diagnosis of generalized dystonia was considered due to the alterations of the neurologic development, and secondary causes were therefore studied.

Routine laboratory evaluation including ceruloplasmin, ferritin, iron, copper, lipid profile, and antinuclear antibodies were within normal ranges. Peripheral blood film did not reveal acanthocytes. Doppler sonography of brain parenchyma was normal, and fundus examination was unremarkable, without Kayser–Fleischer ring on slit-lamp test. Unfortunately, analysis to detect *PANK2* gene mutation could not be done in our country. Both lactic acid (6.6 mg/dl, normal range 4.5–19.8 mg/dl) and serum cooper (100 µg/dl, normal range 80–156 µg/dl) were within normal limits.

**Fig. 1:**
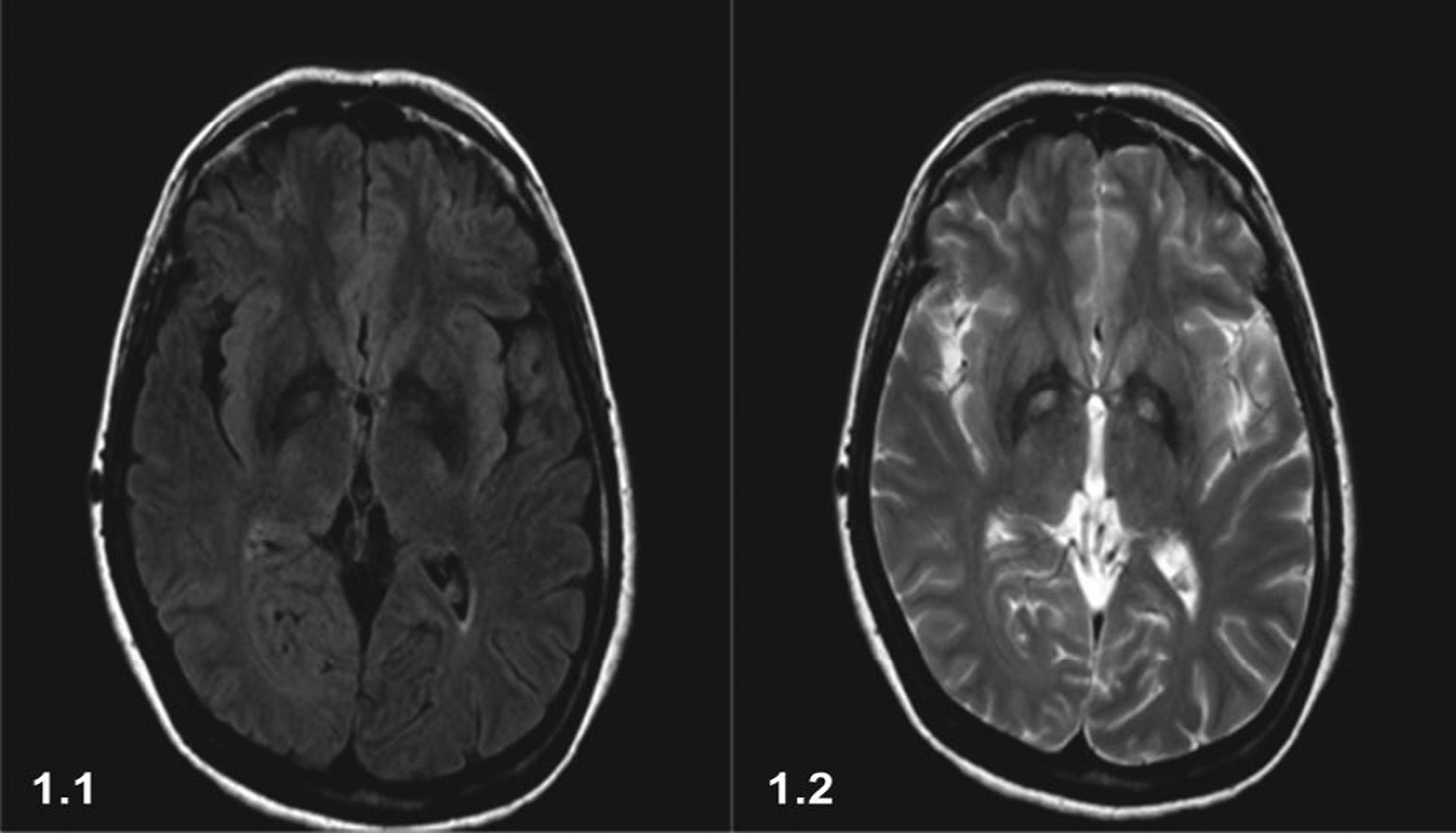
MRI brain showing T2 axial (1.1) and FLAIR (1.2) images: bilateral symmetrical hypointensity in globus pallidus with central hyperintensity “eye of the tiger” sign, due to pantothenate deficit

Cerebral MRI showed bilateral low-density globus pallidus with soft central high-intensity zones in T2 and fluid-attenuated inversion recovery (FLAIR) sequences. The observations were also present in ferromagnetic susceptibility sequences, matching with the “eye of the tiger” sign, compatible with iron deposits and globus pallidus damage (Fig 1).

Wilson’s disease, neuroacanthocytosis, and the juvenile form of Huntington disease were ruled out. In addition, despite the lack of genetic tests, all subtypes of NBIA listed in Table 1 [5] were also considered.

**Table 1:** NBIA subtypes described to date and mode of inheritance

Autosomal recessive
Pantothenate kinase-associated neurodegeneration (PKAN)
Phospholipase A2-associated neurodegeneration (PLAN)
Mitochondrial membrane protein-associated neurodegeneration (MPAN)
Fatty acid hydroxylase-associated neurodegeneration (FAHN)
Coenzyme A synthase protein-associated neurodegeneration (CoPAN)
Kufor Rakeb syndrome
Woodhouse–Sakati syndrome
Aceruloplasminemia
Autosomal dominant
Neuroferritinopathy
X-linked dominant
Beta-propeller protein-associated neurodegeneration (SPAN)

From: Nassif D, Pereira JS, Spitz M, Capitao C, Faria A. Neurodegeneration with brain iron accumulation: a case report. *Dement Neuropsychol*. 2016; 10(2):160–164. https://doi.org/10.1590/S1980-5764-2016DN1002014

Diagnosis of severe type 1 NBIA, based on neurological symptoms/signs as well as MRI findings, was made. Both symptomatic treatment and physiotherapy were started, with cabergoline (1 mg) one tablet each 5 days, trihexyphenidyl (5 mg) one tablet each 8 hours, baclofen (10 mg) one tablet each 8 hours, and intramuscular botulinum neurotoxin (100 U/4 ml) 25 U in the latissimus dorsi, trapezius, sternocleidomastoid, and brachial triceps muscles, bilaterally, each 3 months. Besides, the patient underwent two daily home sessions of passive stretching, weight bearing, and muscle massaging performed by his mentors supervised by local healthcare givers. In addition, a healthy diet based on fruit and vegetable formulae strengthened with meat was prescribed. At 3 months reevaluation, the patient showed great improvement of motor function with decreased dystonic symptoms, although language, cognition, and functional independence showed no improvement.

## Discussion

Brain iron accumulation was first reported in 1922 by Julius Hallervorden and Hugo Spatz, two German neuropathologists who named the disease. Unfortunately, they conducted unethical studies on brain specimens of mentally handicapped persons, executed during the Third Reich “Racial Hygiene” program; therefore, the term HSD has progressively entered disuse, being substituted by type 1 NBIA or PKAN [6], distinguished from other subtypes of NBIA by later onset during childhood, MRI findings, laboratory tests, and genetic analysis [7].

Our patient’s diagnosis was made on the basis of clinical and MRI findings. Genetic analysis could not be done in our country, which is an objective limitation. The age of the patient at onset was 5 years, when he started to present gait alterations and dystonic postures. Marshall *et al*. obtained in their study a median age of PKAN symptom onset of 7.0 years, with a wide range (< 1.0–20.0 years). They also referred that nearly 70.0% of families reported that patients had problems with walking as a presenting symptom, and that high-severity PKAN presented uniformly with walking difficulties [4]. This information coincides with the current report.

In terms of MRI findings, the patient’s T2 and FLAIR sequences showed globus pallidus changes, described by Sethi *et al*. as the “eye of the tiger” sign, seen only in NBIA [8]. Initially, hyperintense areas are seen at globus pallidus and substantia nigra. As NBIA progresses, a hypointense rim is seen around it, due to iron deposition. The evolution of PKAN suggests that neuroaxonal degeneration is followed by pallido-nigral pigmentation and deposition, which is only a late phenomenon. The hyperintensity represents pathologic changes, including gliosis, demyelination, neuronal loss, and axonal swelling, while the surrounding hypointensity is due to loss of signal secondary to iron deposition [2]. From onset to date of diagnosis, 14 years have passed. The average survival after diagnosis is about 12 years due to progression of symptoms [6]. We found only two case reports from Cuba, describing three different patients diagnosed during childhood with early stages of the disease, none with the symptomatic severity seen in our patient [9, 10].

Once the diagnosis was established, treatment was indicated. Treatment of patients with NBIA remains directed toward symptomatic findings. Hypertonia is usually a combination of rigidity and spasticity and may be difficult to treat. Anticholinergic agents may help to reduce motor symptoms. Baclofen and trihexyphenidyl remain the most effective drugs for disabling dystonia and spasticity. When oral baclofen is no longer able to adequately control the movement disorder, placement of a continuous intrathecal baclofen pump may be considered [3]. In addition, botulinum toxin as a blocker of acetylcholine release can be helpful for many patients, especially those whose quality of life is improved by treating a limited body region. Tremor responds best to dopaminergic agents such as cabergoline or levodopa [11]. Other drugs, although not available in Cuba, have proved efficacy to treat the symptoms. The anticholinergic agent benztropine helps rigidity and tremor [3]. Benzodiazepines have been tried for choreoathetotic movements. Dementia is progressive, and no treatment has been proved to be clearly effective [11]. Symptoms such as drooling and dysarthria can be troublesome, but have been efficiently treated with methscopolamine bromide, oral glycopyrrolate, and hyoscine patch [3], although none of these drugs are available in Cuba. Dysarthria may also respond to medications used for rigidity and spasticity. Speech therapy may also be useful, and computer-assisted devices may be employed in the treatment of advanced patients. Gastrostomy feeding may be necessary in advanced cases of dysphagia [11]. Furthermore, correct dietetic advice, rich in fruits and vegetables with a proper vitamin and mineral supply, may also be helpful to create an alkaline balance to decrease brain iron accumulation [9]. Currently, new treatment strategies have been introduced with promising outcomes. Bilateral globus pallidus internus deep brain stimulation has been successful in refractory generalized dystonia in PKAN patients. Dystonia severity assessment using the Burke–Fahn–Marsden Dystonia Rating Scale (BFMDRS) at 1 month demonstrated a 31.3% improvement on the motor scale score [12]. Stimulating another target, such as subthalamus, could also be considered as a possible alternative in this condition [13].

Regular physiotherapy for normalization of muscle tone and improving functional independence is necessary. Passive stretching, reflex inhibiting postures, weight bearing, sensory stimulation, physio-ball therapy, and many more approaches help such patients to regain some functional skills and improve quality of life [3]. In general terms, a multidisciplinary approach and constant evaluation and medication of symptoms must always be performed to slow the progression of the disease and increase patient quality of life.

## Conclusion

The diagnosis of PKAN can be made based on the presence of clinical and imaging features. The presence of “eye of the tiger” sign on MRI due to iron accumulation in globus pallidus must be considered as a nearly pathognomonic sign of the presence of NBIA. Treatment after high-severity presentation remains directed toward symptomatic findings. Both dopamine agonist and anticholinergic agents are useful to treat motor symptoms, but there is not yet an effective treatment to stop the underlying degeneration. New therapeutic approaches are needed to counteract late stages of the disease and improve prognosis.

## Data Availability

The data that support the findings of this study are available from the corresponding author on request.

## Abbreviations

MRI: Magnetic resonance imaging
PKAN: Pantothenate kinase-associated neurodegeneration
HSD: Hallervorden–Spatz disease
NBIA: Neurodegeneration with brain iron accumulation
